# Strong temporal variation of consumer δ^13^C value in an oligotrophic reservoir is related to water level fluctuation

**DOI:** 10.1038/s41598-023-30849-9

**Published:** 2023-03-04

**Authors:** Lukáš Veselý, Fabio Ercoli, Timo J. Ruokonen, Martin Bláha, Jindřich Duras, Phillip J. Haubrock, Martin Kainz, Heikki Hämäläinen, Miloš Buřič, Antonín Kouba

**Affiliations:** 1 Faculty of Fisheries and Protection of Waters, South Bohemian Research Centre of Aquaculture and Biodiversity of Hydrocenoses, University of South Bohemia in České Budějovice, Zátiší 728/II, 389 25 Vodňany, Czech Republic; 2 Department of Biological and Environmental Science, University of Jyväskylä, P.O. Box 35, 40014 Jyväskylä, Finland; 3 Institute of Agricultural and Environmental Sciences, Chair of Hydrobiology and Fishery, Estonian University of Life Sciences, Kreutzwaldi 5, 51006 Tartu, Estonia; 4 Natural Resources Institute Finland, Survontie 9 A, 40500 Jyväskylä, Finland; 5 WasserCluster Lunz – Biological Station, Dr. Carl Kupelwieser Promenade 5, 3293 Lunz Am See, Austria; 6 Department of Biomedical Research, Danube University Krems, Dr. Karl Dorrek-Straße 30, 3500 Krems, Austria; 7 Department of River Ecology and Conservation, Senckenberg Research Institute and Natural History Museum Frankfurt, Clamecystr. 12, 63571 Gelnhausen, Germany; 8 CAMB, Center for Applied Mathematics and Bioinformatics, Gulf University for Science and Technology, Mubarak Al-Abdullah, Kuwait

**Keywords:** Community ecology, Ecosystem ecology, Freshwater ecology, Stable isotope analysis

## Abstract

Using stable carbon and nitrogen isotope analysis (δ^13^C and δ^15^N) to assess trophic interactions in freshwater ecosystems is a well established method, providing insight into ecosystem functioning. However, the spatial and temporal variability of isotope values, driven by environmental fluctuation is poorly understood and can complicate interpretations. We investigated how the temporal variation of stable isotopes in consumers (fish, crayfish and macrozoobenthos) of a canyon-shaped oligotrophic reservoir is associated with environmental factors such as water temperature, transparency, flooded area, and water quality measures. Consumers and their putative food sources were sampled and analyzed for carbon and nitrogen stable isotopes annually, and environmental parameters were measured monthly from 2014 to 2016. Results revealed significant differences in δ^13^C and δ^15^N values in each consumer among studied years. Over the years, fish and crayfish expressed differences in δ^13^C between 3 and 5‰, whereas in zoobenthos differences were 12‰. Variability in δ^15^N was similar across all consumers (2–4‰). Moreover, results suggest that the flooded area of the reservoir was a major driver of δ^13^C stable isotope values variation in consumers, while variation in δ^15^N was not linked to any of the studied environmental factors. Bayesian mixing models further showed significant changes in the origin of detritivorous zoobenthos carbon sources (reversal shift from terrestrial detritus to algae origin) between years with low water level to years with the standard water level. Other species showed only slight differences in food source utilization among years. Our study highlights the importance of environmental factors as sources of variation in consumer’s stable isotope values which should be considered especially when studied ecosystem strongly fluctuate in some environmental factor.

## Introduction

Stable isotopes analyses (SIA) are widely used for determining consumer resource utilizations and interactions in a wide variety of ecostems^1^. It is applicable to a variety of aquatic organisms from all trophic levels^2^, providing insights into aquatic food webs^1^. Among other applications, SIA can be used to estimate the relative importance of consumed autochthonous and allochthonous sources of organic matter^3^, and can detect long-term feeding interactions^4^. Nevertheless, each method has its limitations^5^ and variability in consumer stable isotopes values due to external drivers (i.e. in environmental variables such as water quality, habitat availability, and surface runoff from catchment) may cause uncertainty for interpretation of SIA results.

Temporal and spatial variability in consumer stable isotopes values are well-known in aquatic ecosystems^2,6,7^ and might reflect changes in adjacent ecosystems^8–10^. Values of stable carbon and nitrogen isotopes (δ^13^C and δ^15^N) of consumers generally reflect dietary carbon and nitrogen source intakes from past weeks to few months, depending on species, size, physiological processes, food availability, and temperature^2^. However, there is a substantial lack of information, linking variability in both aquatic consumer δ^13^C and δ^15^N values to changes in environmental variables over time or between ecosystems. Thus, such lack of information might lead to biases in long term (multiple year sampling) data interpretation from given ecosystems. Therefore, studies disentangling environmental factors and stable isotopes values changes are urgently needed.

Several paleoclimate studies suggest that stable isotopes values might reflect past large-scale environmental changes (i.e. climatic changes^11–13^). Yet, there is a lack of information linking changes in consumer stable isotopes values from natural conditions to environmental variables in time and space over shorter time spans. This informational knowledge gap might limit or create a bias in consumer-resource data interpretation in rapidly changing aquatic ecosystems.

Generally, spatial heterogeneity of consumer stable isotopes values is expected from large aquatic ecosystems^14^, possibly originating from a wide variety of available food sources or specific feeding preferences ^15,16^. For instance, Devlin et al.^8^, Ruokonen et al.^17^, and Veselý et al.^18^ have shown that the food source utilization of consumers may vary along the depth gradient of a waterbody, causing intraspecific variation in stable isotope values at the population level.

Temporal variation can be divided into seasonal and annual variability^19^. The former is created by seasonally changing consumer resource utilization and therefore reflects changes in food source availability, isotopic basal source variation, as well as physiological processes in the consumer body^18,20–23^. Although seasonal variability in consumer stable isotope values can be extensive, the magnitude of annual variation is usually low, making these temporal changes less visible at higher trophic levels within a given aquatic food web^18,24^. Still, an extensive change in stable isotopes inter-annual variation might appear, usually reflecting strong changes in the given ecosystems.

Therefore, it is important to reveal the reasons underlying stable isotopes inter-annual variation. Here, we analyzed annual changes in stable isotopes of carbon and nitrogen values of consumers and assessed drivers of variation in the canyon-shaped reservoir Nýrsko, Czech Republic. We hypothesized that (i) food sources are utilized by consumers differently over the years due to variations in environmental factors (temperature, transparency, flooded area, oxygen, pH, NH_4_^+^, chemical oxygen demand by manganese – COD_Mn_, and Chlorophyl *a*). Thus, (ii) the values of stable isotopes differ among consumers across years and within years due to different functional traits born by the species. This study will connect environmental variables to consumer stable isotope values variation over three years. Such results are of interest for food web ecologists and paleobiologists using stable isotopes as a proxy of environmental changes.

## Material and methods

### Study site

Nýrsko reservoir is an oligotrophic water supply reservoir (A = 1.48 km^2^, max. depth = 34 m) in the West Bohemia region of the Czech Republic (49°15′27″N, 13°8′46″E). The left side of the canyon-shaped reservoir gradually slopes to the bottom, which is composed of fine particles and covered by macrophytes. The right side of the reservoir is steeper, with stony shores in its lower section and a mix of sand and stony spots in its middle section. Muddy bottoms form the inlet section of the reservoir on both shores. Seasonal water level fluctuation between 2014 and 2016 was marginal, not exceeding 0.5 m and the flooded area ranged between 1.27 and 1.31 km^2^. In 2015, there was a planned dam reconstruction: water level decreased by 1.5 m below the normal operating level in late April—with water level fluctuation reaching 0.7 m at maximum. The flooded area ranged between 1.24 and 1.25 km^2^.

### Field sampling

All major biological components were sampled annually from 2014 to 2016 in the first week of August. The consistent sampling protocol of Veselý et al.^18^ was applied. We sampled the same species groups over the years. Fish were collected by angling and gillnetting; the full list of species can be found in Veselý et al.^18^. For this study, adult European perch *Perca fluviatilis* L. 1758 and adult roach *Rutilus rutilus* (L. 1758) were chosen due to their presence in sufficient numbers in each year of sampling. Adult noble crayfish *Astacus astacus* (L. 1758) were caught manually with handheld nets as well as by scuba diving and with baited traps along the shoreline in the late afternoon and collected the following morning. Bulk crustacean zooplankton samples were collected using a net (mesh size 250 µm) pulled vertically through the water column. Zoobenthos was collected down to 1 m depth using a hand net (mesh size 500 µm). Macrophytes, periphyton and terrestrial detritus were collected by hand from the shoreline. All samples were placed on dry ice immediately after collection and then transferred to the laboratory freezer (− 30 °C) until further processing for SIA of carbon (δ^13^C) and nitrogen (δ^15^N). A piece of white dorsal muscle tissue of fish and a piece of abdominal muscle tissue of crayfish were used for SIA as recommended by Stenroth et al.^25^. Samples of fish, zoobenthos, terrestrial detritus, and macrophytes were identified to species or genus level. Zoobenthos species were further assigned into functional groups (Table S1). In general, we sampled same species over the years among groups Environmental data were obtained from the Vltava River Authority (Table 1).

**Table 1:** Mean values of monitored environmental variables of Nýrsko reservoir (Czech Republic) from dam part of reservoir used as predictor variables for linear model with mixed effect.

Variables	Year														
	2014					2015					2016				
	April	May	June	July	August	April	May	June	July	August	April	May	June	July	August
Temperature (°C)	10.3	13.5	19.7	20.8	19	10.6	15.5	17.6	23.6	21.5	10	15.3	18.3	17.1	19.5
Transparency (m)	7.2	7.5	6.9	6.9	7	4.8	5.2	5.5	7.7	9.5	6.5	8	5.9	5.2	5.2
Flooded area (1000 m^2^)	1279	1291	1319	1307	1291	1258	1251	1251	1251	1254	1320	1302	1297	1299	1314
O_2_ (mg/l)	11.1	10.5	9.1	9.6	9.1	11.2	10.1	9.6	8.6	8.7	11	10.2	10.2	9	9.4
pH	7.5	7.4	7.1	6.9	6.6	7.2	7.7	7.6	7.7	7.5	6.7	8.2	8	8.2	7
NH_4_^+^ (mg/l)	0.05	0.04	0.04	0.04	0.07	0.03	0.05	0.03	0.06	0.05	0.03	0.03	0.03	0.03	0.03
COD_Mn_ (mg/l)	1	1.2	1.8	1.6	2	1.8	1.2	1.5	1.6	1.5	1.5	1.5	1.5	2.3	2.1
Chl*a*	2.5	0.5	0.5	1.3	2.4	1.7	3.3	2.5	3.4	2.5	1.6	2.1	1.9	4.4	2.7

*COD*_Mn_ chemical oxygen demand by manganese method, *Chla* Chlorophyl *a.*

All handling with organisms were conducted according to the principles of the Institutional Animal Care and Use Committee (IACUC) of the University of South Bohemia, Faculty of Fisheries and Protection of Waters, Research Institute of Fish Culture and Hydrobiology, Vodňany, based on the EU harmonized animal welfare act of Czech Republic. The entire study were approved by Ethical Commitee (IACUC—Institutional Animal Care and Use Committee). The principles of laboratory animal care and the national laws 246/1992 and regulations on animal welfare were followed (Ref. number 22761/2009-17210). This study is reported in accordance with Arrive guidelines (https://arriveguidelines.org). The noble crayfish were sampled under the permit from the Šumava Protected Landscape Area (Ref. number NPS 03232/2011).

No specific permissions were required for working with plants. Nevertheless, All procedures were conducted according to Czech law and in accordance to the standard guidelines.

### Stable isotope analyses

Before the analysis of stable isotopes, all samples were dried at 50 °C for 48 h to the constant weight and grounded to a fine homogenous powder. Approximately 0.6 mg of animal samples and 1.5 mg of plant and detritus samples were weighed (at the precision of 0.001 mg) and transferred into tin cups. Stable isotope analyses were performed at the University of Jyväskylä using a Carlo Erba Flash EA 1112 elemental analyser connected to Thermo Finnigan DELTAplus and Advantage continuous-flow isotope ratio mass spectrometer (Thermo Electron Corporation, Waltham, MA, USA).

Vienna Pee Dee belemnite and atmospheric N_2_ were used as reference standards for carbon and nitrogen, respectively. To control instrument stability, northern pike *Esox lucius* L., 1758 muscle tissue and birch *Betula pendula* R. leaves of known isotopic compositions were run after every six samples. Results are expressed using the conventional δ notation as parts per thousand difference from the international standards. Analytical precision was < 0.1 ‰ for δ^13^C and < 0.3 ‰ for δ^15^N.

### Statistical analyses

#### Effect of environmental factors on stable isotopes variation

Linear mixed effects models were employed to assess the role of environmental factors as a driver of carbon and nitrogen stable isotopes values fluctuation over the years. For each species or functional group, a separate analysis was performed. Given that the values of stable isotopes in the animal body are gathered over time, we used environmental data from the end of April to the end of July of each respective year. To omit the effects of monthly variability on stable isotopes value, we used month as a random effect. The final model was determined by sequential deletion of the least significant explanatory variables from the full model. Parameter significance was evaluated using F-tests from analysis of deviance. The final model included only parameters with significant p-values. Temperature, transparency, flooded area, oxygen, pH, NH_4_^+^, chemical oxygen demand by manganese (COD_Mn_), and Chlorophyl *a* were used as explanatory variables of stable isotopes values changes (Table 1, Fig. S1). These parameters were chosen using correlation matrix where parameters with high correlation with used parameters were omitted (value 0.6 consider as threshold among parameters used in analyses). The significant parameters of the final model, a simple linear regression was applied to test the biological effect of given environmental variables. We compared the slopes of given variables among consumers using the *lstrends* function of the ‘lsmean’ package and ran generalized linear models to reveal differences in each consumer carbon and nitrogen stable isotopes over the years. For all statistical tests, p-values < 0.05 were considered significant. Analyses were performed in R-software^26^ (4.05).

#### Food source utilization

To quantify the contribution of the different food sources to the isotopic signature of each consumer or functional group of consumers a separate Bayesian mixing model^27^ with a specific number of putative sources was run over years in MixSiar-package^28^ in R^26^. In European perch (n = 21), a four-source model was run (omnivorous fish, crayfish, zoobenthos and crustacean zooplankton; Table S2). In roach (n = 20) and noble crayfish (n = 21), a five-source model was run (zoobenthos, crustacean zooplankton, macrophytes, algae and detritus; Table S3). For predatory zoobenthos (n = 20), a two-source model including crustacean zooplankton and zoobenthos was run (Table S4.). In detritivorous zoobenthos (n = 20), a three-source model was run (macrophytes, algae and detritus, Table S5).

## Results

**Figure 1:**
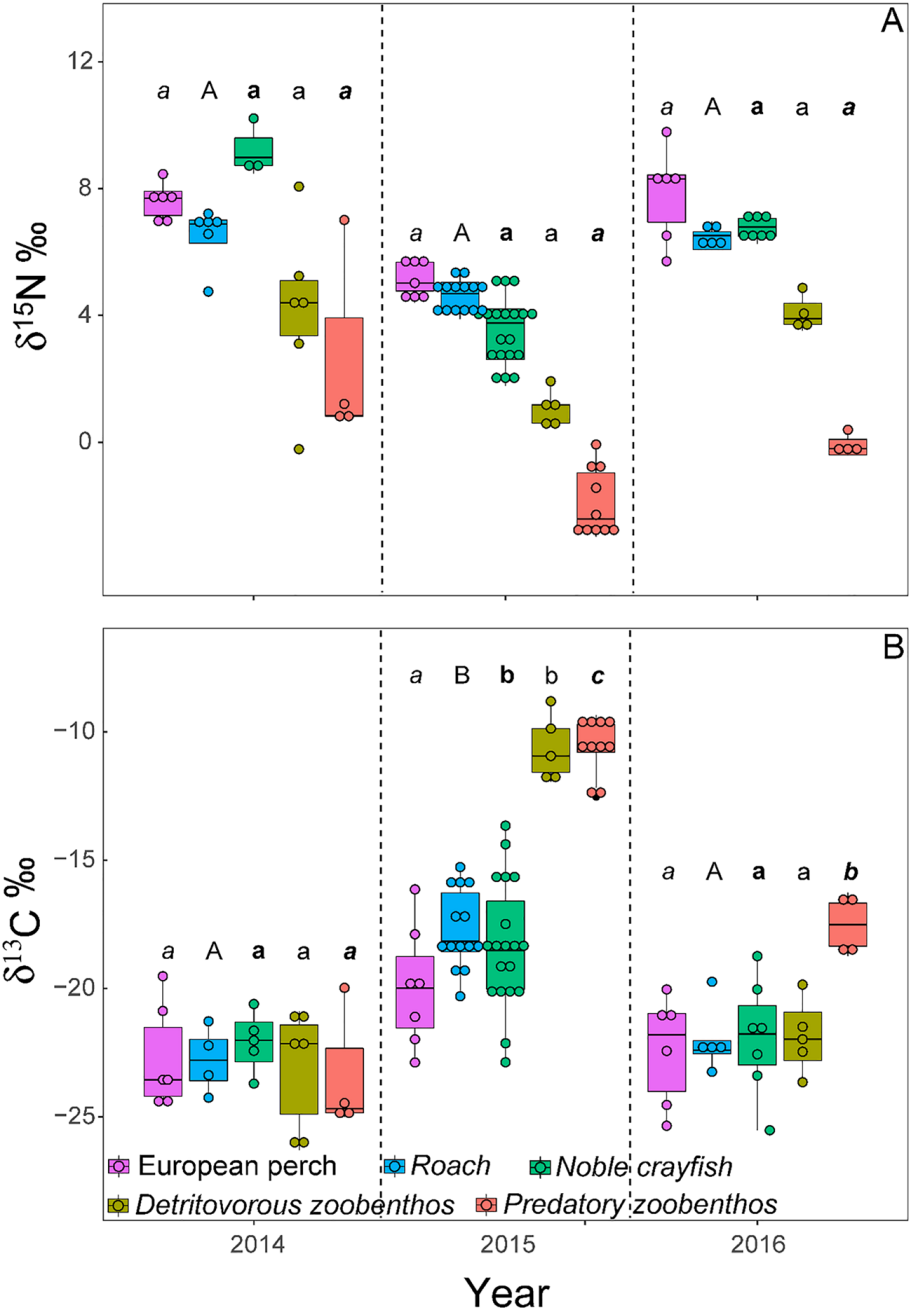
δ^15^N (**A**) and δ^13^C (**B**) values of consumers over time. Letters of significance denote differences over the years within species and functional groups. Perch = small italic letters, Roach = capital letters, noble crayfish = small bold letters, predatory zoobenthos = small letters, detritivorous zoobenthos = bold letters in italic.

Consumer carbon stable isotope values varied across years, albeit the magnitude of variation depending on species and functional group (Fig. 1). In general, δ^13^C and δ^15^N values were similar in 2014 and 2016 although a significant change was noticed in 2015, when the carbon values were enriched across species and groups (Fig. 1B). The highest mean differences in carbon stable isotope values were found in predatory and detritivorous zoobenthos between 2014 and 2015, reaching 12.4 and 12.5‰, respectively. Consumers mean differences between 2014 and 2015 in European perch, roach, and noble crayfish were 2.44, 5.27, and 3.78‰, respectively (Fig. 1B). Annual variation in consumer δ^15^N was much less variation among years was much less pronounced and did not change more than 3‰ (Fig. 1A).

Linear mixed effects models showed that environmental factors were not significantly related to changes of δ^15^N in any of the consumers over the years (p > 0.05). Controversely, δ^13^C stable isotope values of roach, noble crayfish, and detritivorous as well predatory zoobenthos were significantly associated with the size of the flooded area.

**Figure 2:**
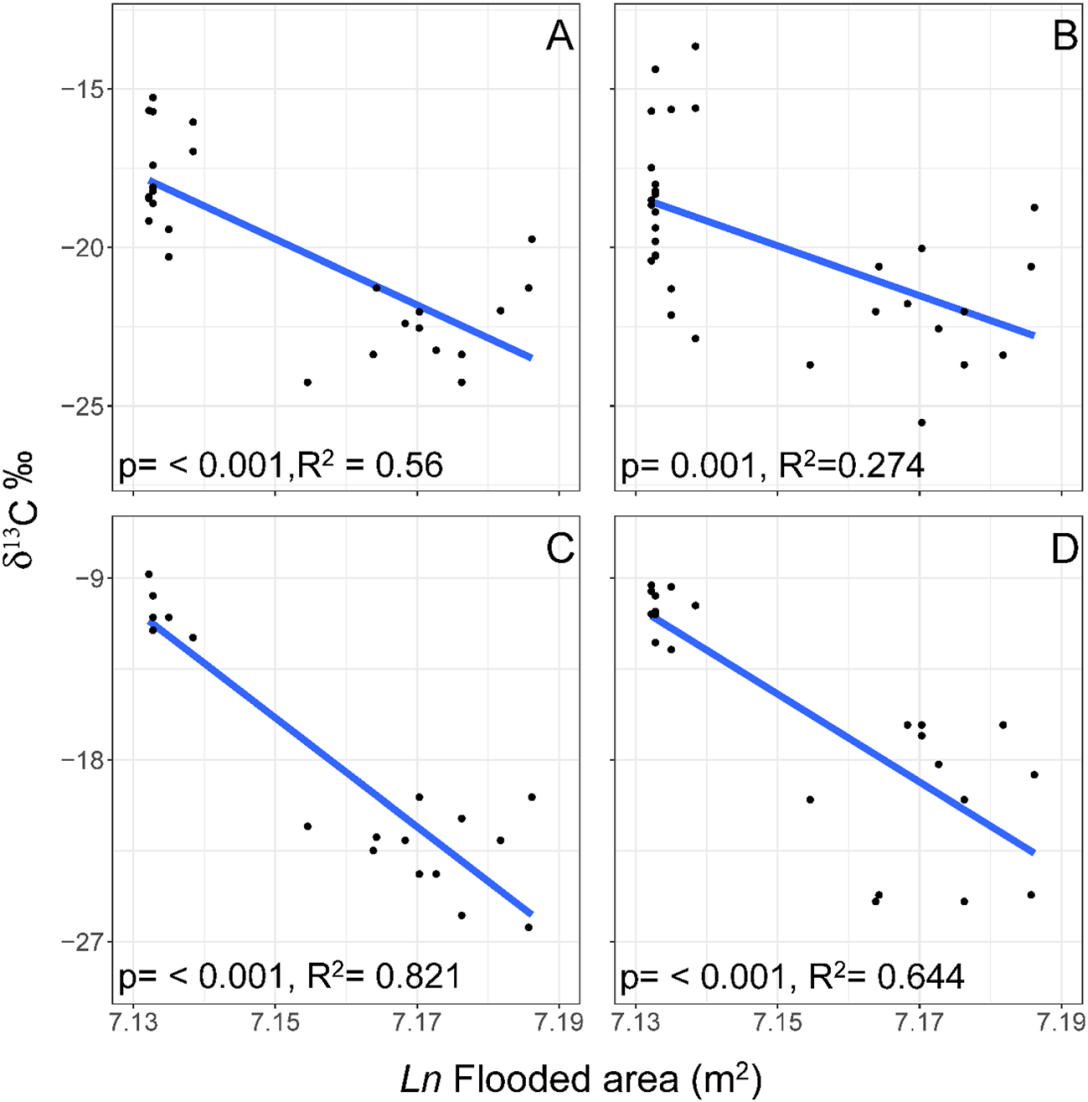
Linear regression of consumers with flooded area in Nyrsko reservoir. (**A**) Roach, (**B**) Noble crayfish, (**C**) Predatory zoobenthos, (**D**) Detritivores zoobenthos.

The extent of the flooded area differed across years, being most reduced in 2015. Neither δ^13^C nor δ^15^N values of the European perch were linked to environmental variables (Table 2). The other environmental variables did not differ over time (Fig. S1). Linear regressions between consumers δ^13^C and the flooded area revealed that with increasing extent of the flooded area, the carbon isotope values of consumers was more depleted (Fig. 2). Differences among slopes of linear regressions suggest specific response of given consumer to extent of the flooded area. Although roach and noble crayfish did not differ in their respective slopes (p = 0.87 and 0.57, respectively), significant differences were found between roach and predatory zoobenthos (p < 0.001 and p = 0.01, respectively), as well as between crayfish and predatory zoobenthos, as well as crayfish and detritivorous zoobenthos (p < 0.001 and p < 0.001, respectively).

**Table 2:** Summary of the most parsimonious model (linear mixed effect model) of δ^13^C for all consumers over the years.

	df	F	*P-value*
Perch			
Temperature	1	0.059	0.812
Transparency	1	0.007	0.934
Flooded area	1	3.730	0.087
O_2_	1	< 0.001	0.995
pH	1	1.820	0.214
NH_4_^+^	1	3.424	0.101
DOC_Mn_	1	0.363	0.560
Chl*a*	1	0.226	0.646
Roach			
Flooded area	1	33.880	** < 0.001**
Crayfish			
Flooded area	1	12.739	** < 0.001**
Predatory Zoobenthos			
Flooded area	1	79.001	** < 0.001**
Detritivorous Zoobenthos			
Flooded area	1	39.138	** < 0.001**

Significant values (P < 0.05) in bold.

*df* degrees of freedom, *COD*_*Mn*_ chemical oxygen demand by manganese method.

Differences in food source utilization were found in all consumers, with the most prominent different seen in 2015 when compared with 2014 and 2016. Changes in food source utilization were consumer-specific (Tables 3, 4, 5 and 6). In 2015, European perch utilized less zoobenthos which was replaced by crustacean zooplankton (Table 3), but used other food sources rather equally over the 3 years. Although zoobenthos was the most important food source for roaches during the study years (Table 4), the roach increased macrophyte utilization during low water levels in 2015. Noble crayfish decreased its utilization of zoobenthos and relied more on crustacean zooplankton and detritus as food sources in 2015 compared to other years (Table 5). Predatory zoobenthos increased zoobenthos and decreased crustacean zooplankton use in 2015 compared to the remaining years (Table 6). In detritivorous zoobenthos, a clear increase in algae use was observed in 2015 (Table 6).

**Table 3:** The relative contribution (median with upper and lower 95% CI (credible intervals) of putative food sources to the diets of European perch in Nýrsko reservoir, Czech Republic.

Food sources	Year	Low 95% CI	Median % contribution	High 95% CI
Omnivorous fish	2014	0.008	0.125	0.354
	2015	0.004	0.155	0.424
	2016	0.005	0.164	0.500
Crayfish	2014	0.007	0.110	0.294
	2015	0.003	0.142	0.527
	2016	0.003	0.138	0.471
Zoobenthos	2014	0.156	0.460	0.719
	2015	0.062	0.274	0.534
	2016	0.108	0.419	0.715
Zooplankton	2014	0.077	0.288	0.528
	2015	0.179	0.371	0.571
	2016	0.027	0.211	0.538

**Table 4:** The relative contribution (median with upper and lower 95% CI (credible intervals) of putative food sources to the diets of roach in Nýrsko reservoir, Czech Republic.

Food sources	Year	Low 95% CI	Median % contribution	High 95% CI
Zoobenthos	2014	0.104	0.422	0.721
	2015	0.056	0.409	0.674
	2016	0.037	0.456	0.884
Zooplankton	2014	0.006	0.105	0.358
	2015	0.003	0.055	0.217
	2016	0.002	0.083	0.406
Macrophytes	2014	0.058	0.255	0.613
	2015	0.124	0.355	0.659
	2016	0.013	0.211	0.761
Algae	2014	0.003	0.069	0.266
	2015	0.001	0.057	0.482
	2016	0.001	0.047	0.405
Detritus	2014	0.003	0.072	0.286
	2015	0.001	0.043	0.210
	2016	0.001	0.045	0.340

**Table 5:** The relative contribution (median with upper and lower 95% CI (credible intervals) of putative food sources to the diets of noble crayfish in Nýrsko reservoir, Czech Republic.

Food sources	Year	Low 95% CI	Median % contribution	High 95% CI
Zoobenthos	2014	0.101	0.573	0.836
	2015	0.080	0.475	0.696
	2016	0.045	0.609	0.944
Zooplankton	2014	0.003	0.080	0.244
	2015	0.002	0.137	0.365
	2016	0.001	0.081	0.325
Macrophytes	2014	0.005	0.132	0.673
	2015	0.003	0.095	0.462
	2016	0.001	0.101	0.768
Algae	2014	0.004	0.091	0.347
	2015	0.002	0.096	0.620
	2016	0.001	0.059	0.429
Detritus	2014	0.003	0.058	0.222
	2015	0.002	0.096	0.367
	2016	0.001	0.034	0.273

**Table 6:** The relative contribution (median with upper and lower 95% CI (credible intervals)) of putative food sources to the diets of predatory and detritivorous zoobenthos in Nýrsko reservoir, Czech Republic.

Food sources	Year	Low 95% CI	Median % contribution	High 95% CI
Predatory zoobenthos				
Zoobenthos	2014	0.398	0.814	0.995
	2015	0.842	0.966	0.998
	2016	0.310	0.875	0.998
Zooplankton	2014	0.005	0.186	0.602
	2015	0.002	0.034	0.158
	2016	0.002	0.125	0.690
Detritivorous zoobenthos				
Macrophytes	2014	0.002	0.052	0.297
	2015	0.000	0.014	0.135
	2016	0.001	0.033	0.483
Algae	2014	0.151	0.385	0.600
	2015	0.667	0.885	0.976
	2016	0.019	0.190	0.733
Detritus	2014	0.348	0.542	0.719
	2015	0.015	0.093	0.266
	2016	0.141	0.726	0.965

## Discussion

This study provides substantial field evidence that long-term water level drops function as a significant driver for the variation in stable isotope values of several consumer groups, exemplary identified in the case of the studied Nýrsko reservoir. This has severe implications for studies dealing with ecosystems with water level fluctuation. Specifically, without knowledge of magnitude of water level fluctuation, comparison with similar systems or multiple year observation might be problematic. In addition, neither hypothesis 1 (food sources are utilized by consumers differently over the years due to variations in environmental factors) nor hypothesis 2 (the values of stable isotopes differ among consumers across years and within years) can be rejected.

The water level of reservoirs fluctuates more frequently than in natural lakes^19,29^, with water level decreases reaching several meters inevitably causing major stress for littoral biota^30^. We presume that in the Nýrsko reservoir, due to major water level drop in 2015, the organic matter from the shallow part of the reservoir, remained almost unreachable to detritivorous zoobenthos. Such stress can result in changes of trophic subsidy from terrestrial (detritus) to aquatic ecosystem (benthic algae) derived carbon δ^13^C in detrivorous zoobenthos. In such cases, detritivorous zoobenthos is obligated to use food sources from deeper zones (i.e. benthic algae), which was emphasized in a clear change from detritus to algae as primary food source (Tb. 6). This change in food source utilization probably caused the observed variation of δ^13^C values of zoobenthos (mean algae and detritivorous zoobenthos δ^13^C value in 2015 was − 14.08 ± 3.26 and 10.55 ± 1.16, respectively), shifting from depleted to enriched values in δ^13^C. This substantial alteration was lifted to higher trophic levels via the trophic chain, likely decreasing the trophic position of noble crayfish by almost two levels downwards. Similarly, to a series of other studies^31–33^, water level drop was found to significantly decrease the density of benthic macroinvertebrates in 2015. Changes observed in the Nýrsko reservoir highlight the instability of such systems and reflect the importance of connecting environmental factors (water level fluctuation in the case of this study) with biomarkers such as bulk stable isotopes. Moreover, observations over multiple years and sites are needed to elucidate the key effects of specific environmental drivers on consumer-specific values. Such results will lead to significantly lower biases in food source utilization models across space or time.

Changes on the lower trophic level were particularly prominent, being known to express staggering trophic fluctuations in accordance with i.e. nutrient influx or depletion^19^. Indeed, zoobenthos was an important food source for consumers in the Nýrsko reservoir, where strong changes in its δ^13^C value in 2015 affected other consumer values. Of course, zoobenthos was not the only food source of consumers as they prey upon many sources. Thus, the values of a given consumer consists of the biomass ratio of given food sources^2^ and concomitantly, a consumer’s response to basal stable isotope changes might vary. Importantly, other physiological factors such as growth rate, varying trophic discrimination factors among consumers and prey, or the time needed by consumers to assimilate new values from a given diet should be taken into account^2,34,35^. Hence, our Bayesian mixing model results suggest that consumers (European perch, roach, and noble crayfish) responded differently to changes in basal food sources, which can be possibly given by their bodies’ different physiological processes and the ecological roles of consumers in the reservoir’s food web.

## Conclusion

Our study demonstrates the importance of environmental factors as a source of variation in the stable isotope values of consumers. Specifically, we found that water level fluctuation among years was the major driver of δ^13^C isotope value changes. Our results therefore suggest that such changes were caused by the inaccessibility of the littoral zone for basal consumers. Nonetheless, not all consumers’ δ^13^C values were related to water level fluctuations, as consumers with higher trophic positions were enriched to a lesser extent, while zoobenthos consumers and predators showed the largest variation in δ^13^C values. Specific responses of a given consumer or its functional group in terms of δ^13^C were related to variability in food source use and likely to differences in their physiological processes and ecology. Moreover, our results highlight the need to jointly test long-term isotopic data and environmental variables of a given aquatic ecosystem to confirm drivers of variability in stable isotope values.

## Supplementary Information

Supplementary Information.

## Data Availability

The datasets used and/or analyzed during the current study available from the corresponding author on reasonable request.
