# Supplementary material for: Strong temporal variation of consumer δ13C value in an oligotrophic reservoir is related to water level fluctuation
Source: Sci Rep. 2023 Mar 4;13:3642. doi: 10.1038/s41598-023-30849-9 (PMC9985621; doi:10.1038/s41598-023-30849-9)
Supplement: Supplementary file 1 — Supplementary Information. [file 41598_2023_30849_MOESM1_ESM.docx]

**Strong temporal variation of consumer δ13C signal in an oligotrophic reservoir is related to water level fluctuation**

Lukáš Veselý^1,5^; Fabio Ercoli^2,3^; Timo J. Ruokonen^2,4^; Martin Bláha^1^; Jindřich Duras^1^; Martin Kainz^5,6^ ; Miloš Buřič^1^; Antonín Kouba^1^

*^1^ – University of South Bohemia in České Budějovice, Faculty of Fisheries and Protection of Waters, South Bohemian Research Centre of Aquaculture and Biodiversity of Hydrocenoses, Zátiší 728/II, 389 25 Vodňany, Czech Republic*

*^2^ – University of Jyväskylä, Department of Biological and Environmental Science, P.O. Box 35, FI-40014 Finland*

*^3^ – Estonian University of Life Sciences, Institute of Agricultural and Environmental Sciences, Chair of Hydrobiology and Fishery, 51006, Kreutzwaldi 5, Tartu, Estonia;*

*^4^* – *Natural Resources Institute Finland, Survontie 9 A, 40500 Jyväskylä, Finland;*

*^5^ – WasserCluster Lunz – Biological Station, Dr. Carl Kupelwieser Promenade 5, 3293 Lunz am See, Austria*

*^6^ – Danube University Krems, Department of Biomedical Research, Dr. Karl Dorrek-Straße 30, 3500 Krems, Austria*

Corresponding author: veselyl@frov.jcu.cz (Lukáš Veselý)

| **Functional group** | **Species** |
| --- | --- |
| **Predatory zoobenthos** | *Erpobdella octoculata* |
|  | *Helobdella stagnalis* |
|  | *Lacophilus hyalinus* |
|  | *Nebrioporus elegans* |
|  | *Sympetrum* sp. |
|  | *Hemiopis sanguisuga* |
|  | *Hygrotus versicolor* |
|  | *Nepa cinerea* |
| **Detritivorous zoobenthos** | *Ceanis* sp. |
|  | *Cloeon dipterum* |
|  | *Ephemera vulgata* |
|  | *Chironomidae* |
|  | *Oligochaeta* |
|  | *Tabanidae* sp. |

**Table S1.** Composition of macroinvertebrates functional groups used in stable isotope analysis.

| **Food sources** | **Years** | | | | | | |
| --- | --- | --- | --- | --- | --- | --- | --- |
|  | **2014** | | | **2015** | | **2016** | |
|  | **δ^13^C** | **δ^15^N** | **δ^13^C** | | **δ^15^N** | **δ^13^C** | **δ^15^N** |
| **Omnivorous fish** | -23.74 ± 1.17 | 7.56 ± 1.14 | -18.87 ± 1.82 | | 4.35 ± 0.57 | -22.57 ± 1.23 | 6.61 ± 0.46 |
| **Noble crayfish** | -22.11 ± 1.55 | 9.22 ± 0.89 | -18.33 ± 2.48 | | 3.51 ± 1.06 | -21.90 ± 2.22 | 6.76 ± 0.36 |
| **Zoobenthos** | -22.40 ± 4.33 | 3.61 ± 2.33 | -14.87 ± 3.79 | | 0.43 ± 1.83 | -21.71 ± 4.26 | 2.44 ± 2.19 |
| **Zooplankton** | -28.01 ± 0.14 | 2.56 ± 0.68 | -25.63 ± 2.10 | | 0.10 ± 0.94 | -27.19 ± 0.14 | 3.78 ± 0.78 |

**Table S2**. The isotopic value of putative food sources of European perch over the years, mean ± SD.

| **Food sources** | **Years** | | | | | | |
| --- | --- | --- | --- | --- | --- | --- | --- |
|  | **2014** | | | **2015** | | **2016** | |
|  | **δ^13^C** | **δ^15^N** | **δ^13^C** | | **δ^15^N** | **δ^13^C** | **δ^15^N** |
| **Zoobenthos** | -22.40 ± 4.33 | 3.61 ± 2.33 | -14.87 ± 3.79 | | 0.43 ± 1.83 | -21.71 ± 4.26 | 2.44 ± 2.19 |
| **Zooplankton** | -28.01 ± 0.14 | 2.56 ± 0.68 | -25.63 ± 2.10 | | 0.10 ± 0.94 | -27.19 ± 0.14 | 3.78 ± 0.78 |
| **Macrophytes** | -19.44 ± 3.51 | 3.83 ± 1.91 | -21.51 ± 6.53 | | 4.79 ± 3.21 | -23.47 ± 7.17 | 3.65 ± 2.04 |
| **Periphyton** | -12.23 ± 3.07 | 0.36 ± 1.31 | -14.08 ± 3.26 | | 0.18 ± 0.58 | -26.43 ± 0.77 | 4.40 ± 2.51 |
| **Detritus** | -28.05 ± 1.33 | -0.84 ±1.68 | -26.87 ± 1.06 | | 0.56 ± 1.69 | -24.84 ± 5.79 | 0.96 ± 0.98 |

**Table S3.** The isotopic value of putative food sources of roach and noble crayfish over the years, mean ± SD.

**Table S4. The isotopic value of putative food sources of predatory zoobenthos over the years, mean ± SD.**

| **Food sources** | **Years** | | | | | | |
| --- | --- | --- | --- | --- | --- | --- | --- |
|  | **2014** | | | **2015** | | **2016** | |
|  | **δ^13^C** | **δ^15^N** | **δ^13^C** | | **δ^15^N** | **δ^13^C** | **δ^15^N** |
| **Zoobenthos** | -19.53 ± 4.74 | 2.53 ± 2.31 | -14.70 ± 3.99 | | -0.57 ± 1.50 | -21.17 ± 4.61 | 1.66 ± 1.38 |
| **Zooplankton** | -28.01 ± 0.14 | 2.56 ± 0.68 | -25.63 ± 2.10 | | 0.10 ± 0.94 | -27.19 ± 0.14 | 3.78 ± 0.78 |

**Table S5. The isotopic value of putative food sources of detritivores zoobenthos over the years, mean ± SD.**

| **Food sources** | **Years** | | | | | | |
| --- | --- | --- | --- | --- | --- | --- | --- |
|  | **2014** | | | **2015** | | **2016** | |
|  | **δ^13^C** | **δ^15^N** | **δ^13^C** | | **δ^15^N** | **δ^13^C** | **δ^15^N** |
| **Macrophytes** | 19.44 ± 3.51 | 3.83 ± 1.91 | 21.51 ± 6.53 | | 4.79 ± 3.21 | -23.47 ± 7.17 | 3.65 ± 2.04 |
| **Periphyton** | -12.23 ± 3.07 | 0.36 ± 1.31 | -14.08 ± 3.26 | | 0.18 ± 0.58 | -26.43 ± 0.77 | 4.40 ± 2.51 |
| **Detritus** | -28.05 ± 1.33 | -0.84 ±1.68 | -26.87 ± 1.06 | | 0.56 ± 1.69 | -24.84 ± 5.79 | 0.96 ± 0.98 |


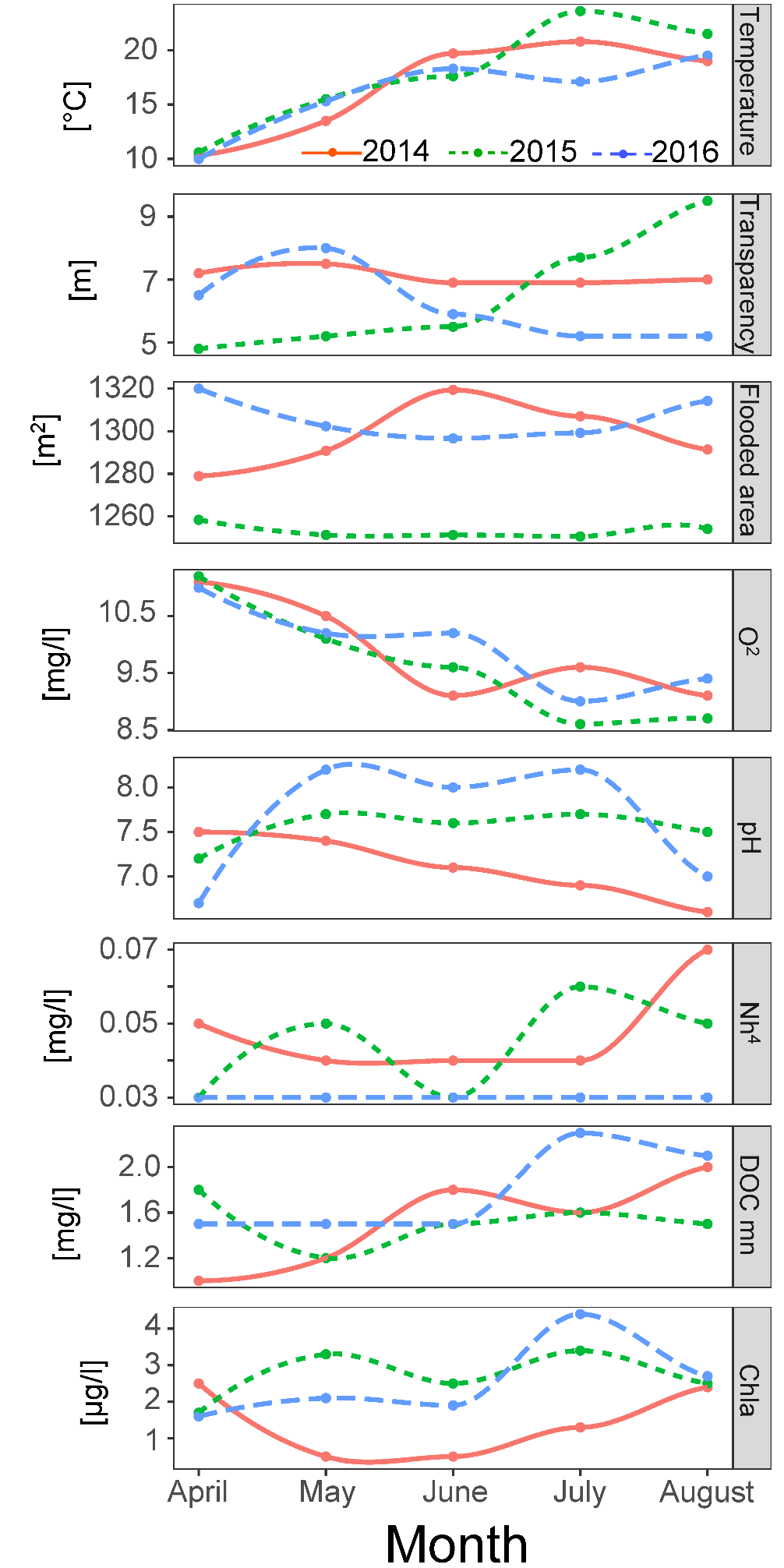


**Figure S1**. Given environmental variables of Nýrsko reservoir (Czech Republic) used as predictor variables for Linear model with mixed effect. COD mn = chemical oxygen demand. Chl*a =* Chlorophil *a*
